# Investigation of the *In Vitro* and *In Vivo* efficiency of RM-532-105, a 17β-hydroxysteroid dehydrogenase type 3 inhibitor, in LAPC-4 prostate cancer cell and tumor models

**DOI:** 10.1371/journal.pone.0171871

**Published:** 2017-02-09

**Authors:** Lucie Carolle Kenmogne, Jenny Roy, René Maltais, Mélanie Rouleau, Bertrand Neveu, Frédéric Pouliot, Donald Poirier

**Affiliations:** 1 Laboratory of Medicinal Chemistry, CHU de Québec - Research Center (CHUL, T4), Québec, Québec, Canada; 2 CHU de Québec - Research Center, Axe Cancer, Québec, Québec, Canada; 3 Department of Surgery, Faculty of Medicine, Université Laval, Québec, Québec, Canada; 4 Department of Molecular Medicine, Faculty of Medicine, Université Laval, Québec, Québec, Canada; Medizinische Universitat Innsbruck, AUSTRIA

## Abstract

In the fight against androgen-sensitive prostate cancer, the enzyme 17β-hydroxysteroid dehydrogenase type 3 (17β-HSD3) is an attractive therapeutic target considering its key role in the formation of androgenic steroids. In this study, we attempted to assess the *in vivo* efficacy of the compound RM-532-105, an androsterone derivative developed as an inhibitor of 17β-HSD3, in the prostate cancer model of androgen-sensitive LAPC-4 cells xenografted in nude mice. RM-532-105 did not inhibit the tumor growth induced by 4-androstene-3,17-dione (4-dione); rather, the levels of the androgens testosterone (T) and dihydrotestosterone (DHT) increased within the tumors. In plasma, however, DHT levels increased but T levels did not. In troubleshooting experiments, the non-androgenic potential of RM-532-105 was confirmed by two different assays (LAPC-4 proliferation and androgen receptor transcriptional activity assays). The enzyme 5α-reductase was also revealed to be the predominant enzyme metabolizing 4-dione in LAPC-4 cells, yielding 5α-androstane-3,17-dione and not T. Other 17β-HSDs than 17β-HSD3 seem responsible in the androgen synthesis. From experiments with LAPC-4 cells, we fortuitously came across the interesting finding that 17β-HSD3 inhibitor RM-532-105 is concentrated inside tumors.

## Introduction

**Fig 1:**
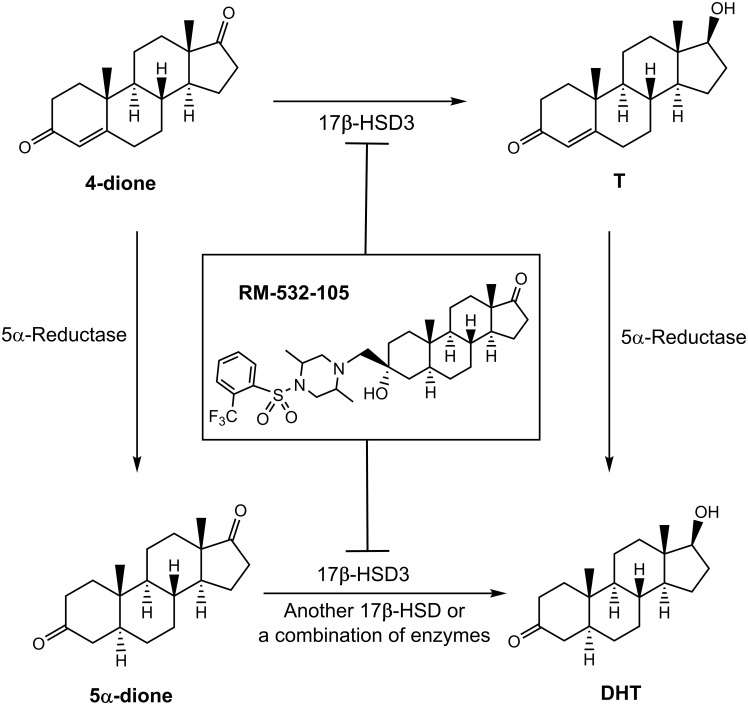
The chemical structure and role of RM-532-105. This steroid derivative inhibits the 17β-hydroxysteroid dehydrogenase type 3 (17β-HSD3), which is involved in the biosynthesis of androgens testosterone (T) and dihydrotestosterone (DHT) from 4-androstene-3,17-dione (4-dione) and 5α-androstane-3,17-dione (5α -dione), respectively.

Despite improvements in cancer therapy development, prostate cancer remains the most diagnosed cancer amongst American and Canadian men. In fact, 1 out of every 7 men will be affected by this cancer during his lifetime [1, 2]. Androgens play a key function in the proliferation of androgen-sensitive prostate cancer [3], and androgen deprivation therapy is the gold standard treatment, either by using an agonist or an antagonist of gonadotropin-releasing hormone (GnRH) alone or an agonist of GnRH in combination with a pure antiandrogen [4–6]. However, given the fact that an important part of active steroids is locally synthesized within prostatic cancer cells (intracrinology) from blood circulating precursors [7, 8], a complementary approach is to develop therapeutic agents that are able to efficiently inhibit the biosynthesis of androgens *per se*. Within the enzymes involved in the biosynthesis of active androgens, we have targeted 17β-hydroxysteroid dehydrogenase type 3 (17β-HSD3). This enzyme catalyzes the last step of the synthesis of active androgen testosterone (T), which is the precursor of dihydrotestosterone (DHT), the most potent androgen in men (Fig 1). 17β-HSD3 is also overexpressed in prostate cancer cells where it is suspected to play a critical role in androgen production [9, 10].

The compound RM-532-105 is an androsterone (ADT) derivative substituted at position 3β that was developed from structure-activity relationship (SAR) studies [11–13] (Fig 1). This steroid derivative bearing an unreactive side-chain is expected to be a reversible inhibitor, but its mechanism of action is not fully known. It has shown *in vitro* 17β-HSD3 inhibitory activity by blocking the transformation of 4-androstene-3,17-dione (4-dione) into T (IC_50_ of 5 nM and 13 nM, respectively) in two experiments involving whole HEK-293 cells and LNCaP cells both overexpressing 17β-HSD3 (HEK-293[17β-HSD3] and LNCaP[17β-HSD3], respectively) [11, 14]. Moreover, when administered subcutaneously to rats *in vivo*, RM-532-105 significantly decreased the plasma levels of T and DHT, 2 h after injection [15].

In this study, we intended to perform *in vivo* proof of concepts by evaluating the activity of RM-532-105 on androgen-stimulated prostate cancer xenografts using prostate cancer LAPC-4 cells. In fact, these cells naturally expressing the wild-type androgen receptor (AR) were selected over the well-known LNCaP cells expressing a mutated AR [16]. We also addressed the effect of this 17β-HSD3 inhibitor on LAPC-4 cell proliferation and 4-dione metabolism.

## Materials and methods

### Cell line and cell culture

Prostate cancer LAPC-4 cells were kindly provided by Robert E. Reiter from the University of California (Los Angeles, CA, USA). They were maintained in exponential growth at 37°C under 5% CO_2_ humidified atmosphere and grown in Iscove's Modified Dulbecco's Medium (IMDM) supplemented (v/v) with 10% fetal bovine serum (FBS), 1% L-glutamine, 1% insulin and 1% penicillin/streptomycin.

### Proliferative activity of RM-532-105 on LAPC-4 cells

Androgen-sensitive LAPC-4 cells were suspended in the culture medium supplemented with 5% dextran-coated charcoal treated FBS rather than 10% FBS. Triplicate cultures of 3,000 cells in a total of 100 μL medium in 96-well microtiter plates (Becton—Dickinson Company, Lincoln Park, NJ, USA) were pre-incubated for 48 h and then washed 4 times to remove the residual hormones. The compound to test was dissolved in ethanol (EtOH) to prepare the stock solution of 10^−2^ M, diluted at several concentrations with experimental medium and added to corresponding wells at the time zero. The final EtOH concentration in each well was adjusted to 0.05%. The medium in wells was changed every 2 days, by removing 50 μL of medium in the well and replacing with fresh medium-containing the required RM-532-105 concentration. After 6 days, quantification of cell growth was determined by 3-(4,5-dimethylthiazol-2-yl)-5-(3-carboxymethoxyphenyl)2-(4-sulfophenyl)-2H-tetrazolium (MTS) method, using CellTitter 96^®^ Aqueous Solution Cell Proliferation Assay (Promega, Nepean, ON, Canada) and following the manufacturer’s instructions. The proliferative activity was expressed as the difference between the cell proliferation (in %) caused by the compound tested and the basal cell proliferation fixed at 100%.

### Androgen receptor transcriptional activity modulation by RM-532-105 in LAPC-4 cells

The androgen-responsive PSEBC promoter was cloned upstream of a two-step transcriptional amplification system (TSTA) and a firefly luciferase reporter gene (fl) in an adenoviral plasmid, as previously described [17]. The adenoviral plasmid was transfected into 293A cells for adenovirus production. Titers were determined using the Adeno-XTM Rapid Titer Kit (Clontech, Mountain View, CA, USA). LAPC-4 cells (1.6 x million/well) were seeded in 24-well plates in culture medium supplemented with 10% charcoal-stripped FBS. Twenty-four hours later, PSEBC-TSTA-fl adenovirus (multiplicity of infection (MOI) of 5) and treatments were added as follows: 1) testosterone (T) (0.1 and 1.0 ng/mL) (Toronto Research Chemicals, Toronto, ON, Canada) was used as a standard curve, or 2) a castrated level of T (0.1 ng/mL) was combined with increasing concentration of RM-532-105 (0.01, 0.1 and 1.0 μM). Each well contained the same volume of vehicle (EtOH). Seventy-two hours after the treatment, cells were lysed and Firefly luciferase activity measured by means of Luminoskan Ascent (ThermoFisher Scientific, Waltham, MA, USA) following the addition of luciferase assay substrate, as stated in the luciferase protocol (Promega, Nepean, ON, Canada). Relative fl activity (RLU) was normalized by the protein content in each well (RLU = RLU ÷ μg of protein). Protein content was estimated by adding 250 μL of Bradford reagent (ThermoFisher Scientific) to 3 μL of total lysate. Absorbance was then read using an Infinite F50 absorbance microplate reader (TECAN, Mannedorf, Switzerland) at 595 nm.

### Metabolism of 4-dione in LAPC-4 cells in the presence or absence of RM-532-105

LAPC-4 cells were plated in 24-well culture at 20,000 cells per well. After incubation for 2 days, 50 nM of [^14^C]-4-androstene-3,17-dione (Perkin Elmer Life Sciences Inc., Boston, MA, USA) and 10 μL of RM-532-105 dissolved in DMSO and culture medium were added. The final DMSO concentration in each was adjusted to 0.05%. After 3 and 5 days, the culture medium was removed from wells and steroids were extracted with diethyl ether. After evaporating the organic phase to dryness with a nitrogen stream, residue was dissolved in dichloromethane, dropped on silica gel 60 F254 thin layer chromatography (TLC) plates (EMD Chemicals Inc., Gibbstown, NJ, USA) and eluted with a mixture of toluene/acetone (4:1). [^14^C]-labeled steroids (4-dione, A-dione, DHT/ADT, epi-ADT and T) were identified by comparison with reference steroids and quantified using the Storm 860 System (Molecular Dynamics, Sunnyvale, CA, USA). The relative abundance of each steroid was calculated and expressed in percentage.

### Animals

We performed *in vivo* experiments under a protocol that was approved by our institutional animal ethics committee (Comités de protection des animaux, Université Laval, Québec, Qc, Canada) and by the Canadian Council on Animal Care. Homozygous male *nu/nu* nude mice (24–42 days old) were purchased from Charles River Inc. (Saint-Constant, QC, Canada) and housed (four to five) in vinyl micro-isolated ventilated cages, equipped with air lids, which were kept in laminar airflow hoods and maintained under pathogen-limiting conditions. During the acclimatization and study period, the animals were housed under a controlled environment at 22 ± 3°C, with 50 ± 20% relative humidity and light set at 12 h/day (light on at 07:15). Sterile rodent food (Rodent diet #T.2018.15, Harlan Teklad, Madison, WI) and water were provided ad libitum.

### Effect of RM-532-105 on LAPC-4 xenografts

Thirty male *nu/nu* nude mice (22–24 g) were assigned in this assay. After the acclimatization period, 24 of them were castrated and the remaining 6 were not, but went through a sham surgery. After receiving 4-dione for 3 days, 24 castrated mice as well as 6 non-castrated mice were inoculated by subcutaneous (s.c.) injection of 10 million LAPC-4 cells (in 0.1 mL of growth medium containing 30% Matrigel (BD Biosciences, Bedford, MA, USA)) into both mouse flanks via a 2.5-cm long 22-gauge needle. After 6 days, the mice were randomly assigned to 5 groups of 6 mice according to their weight (11 to 12 tumors each group).

Two vehicles were used in this experiment, namely vehicle V1 [0.4% aqueous methylcellulose:dimethylsulfoxide (DMSO) (92:8)] and vehicle V2 [propylene glycol (PG):EtOH (90:10)]. Group 1 mice were not castrated but experienced the stress of surgery (SHAM). However, the mice from groups 2, 3, 4 and 5 were castrated (CTX). Group 1 and Group 2 were treated daily with both vehicles V1 and V2. Group 3 was treated daily with 4-dione (200 μg/mouse/0.1 mL in vehicle V2) and received vehicle V1 also. Group 4 was treated daily with 4-dione (200 μg/mouse/0.1 mL in vehicle V2) together with RM-532-105 (50 mg/kg/0.1 mL in vehicle V1). Group 5 was treated daily s.c. with testosterone propionate (TP) (200 μg/mouse/0.1 mL in vehicle V2) and received vehicle V1 also.

Tumor size was measured twice weekly using a caliper. Two perpendicular diameters (*L* and *W*) were measured, and the tumor area (in mm^2^) was calculated using the formula (*L*/2) x (*W*/2) x π. At the end of the xenograft experiment, all the mice were sacrificed 3 h following the last dose. Animals were anesthetized with isoflurane and killed by cervical dislocation. During necropsy, tumors were collected from mice as well as blood by cardiac puncture. Tumor and plasma concentrations of RM-532-105 and of four unconjugated steroids (4-dione, T, DHT and ADT) were determined by LC/MS-MS [15] and GC/MS [18] analyses, respectively, using procedures well established at the CHU de Québec—Research Center (Bioanalytical Service) and previously published [15, 18].

### Statistics

The Duncan-Kramer test was used to analyse the data, and statistical significance accepted at p < 0.05 [19].

## Results

### Effect of RM-532-105 on human LAPC-4 cell proliferation

**Fig 2:**
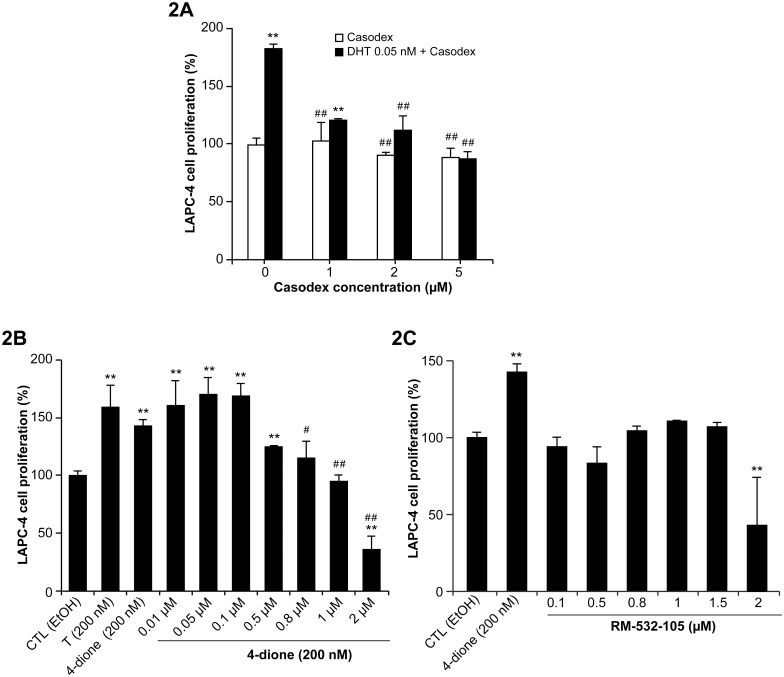
Proliferation of androgen-sensitive LAPC-4 (AR^+^) cells under different treatments. **A)** Effect of antiandrogen Casodex on proliferation induced by DHT. 3,000 cells were pre-incubated for 48 h, washed 4 times and treated 6 days with DHT, a natural potent androgen, and different concentrations (1.0, 2.0, 5.0 and 10 μM of Casodex). Significantly different from the control (CTL) p<0.01 (**) and from DHT + Casodex p<0.01 (##). **B)** Effect of 17β-HSD3 inhibitor RM-532-105 on proliferation induced by 4-dione. 3,000 cells were pre-incubated for 48 h, washed 4 times and treated 6 days with 4-dione, the natural substrate of 17β-HSD3, and different concentrations (0.01, 0.05, 0.1, 0.5, 0.8, 1.0 and 2.0 μM) of RM-532-105. The potent androgen testosterone (T) was used as a positive control for this androgen-sensitive (AR^+^) cell line. Significantly different from the control (CTL) p<0.01 (**) and from 4-dione p<0.01 (##) and p<0.05 (#). **C)** Effect of RM-532-105 alone on cell proliferation. 3,000 cells were pre-incubated for 48 h, then washed 4 times and treated with RM-532-105 for 6 days. Significantly different from the control (CTL) p<0.01 (**).

In prostate cancer research, LNCaP, PC-3 and DU-145 cells are still the most popular for the majority of the published research [20]. These cell lines were however eliminated for our *in vivo* study because they are AR-negative cells (PC-3 and DU-145) or AR-positive cells with a mutated receptor (LNCaP). We then selected LAPC-4 prostate tumor cells since they express a wild-type AR and can form tumors in mice [20]. In culture, we stimulated the LAPC-4 cell proliferation using the potent androgen DHT and blocked this effect by the antiandrogen Casodex (Fig 2A). The cell proliferation was also stimulated by 4-dione at the same level than T, but since 4-dione by itself is not androgenic [10], it must be transformed by LAPC-4 cells into a potent androgen (T or DHT) before mediating its androgenic activity. Interestingly, the effect induced by 4-dione was reversed by a treatment with RM-532-105 (Fig 2B). When RM-532-105 was tested alone (Fig 2C), the LAPC-4 cell proliferation did not increase. In fact, compared to the basal cell proliferation, RM-532-105 shows no significant proliferative androgenic activity at concentrations of 0.1 to 1.5 μM. A cytotoxic activity was however observed at the higher concentration tested of 2 μM.

### Total androgenicity of RM-532-105 on LAPC-4 cells

**Fig 3:**
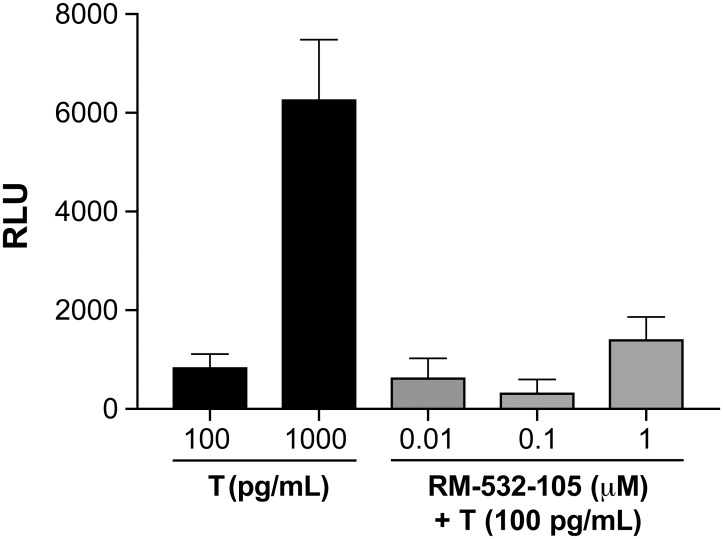
RM-532-105 does not modulate the transcriptional activity of AR *in vitro*. LAPC-4 cells were infected with the PSEBC-TSTA-fl adenovirus (MOI = 5) and treated with testosterone (T) (100 or 1,000 pg/mL). The reporter system is able to quantitatively assess AR activity in LAPC-4 cells, even with castrated (CTX) levels of T (100 pg/mL). The addition of RM-532-105 compound to the CTX level of T (100 pg/mL) does not significantly contribute (p < 0.05) to the transcriptional androgenic activity of LAPC-4 cells. Luciferase assays were performed 72 h post-infection. The relative fl activity (RLU) was normalized by protein content in each well. Wells without T were used as controls for background signal. RLU from control wells were substracted from each treatment. Results are represented as mean ± SD.

PSEBC is a chimeric androgen-responsive promoter derived from the prostate specific antigen (PSA) promoter [21,17]. Previous experiments demonstrated that the transcriptional amplification system PSEBC-TSTA-fl is able to specifically quantify androgen receptor (AR) activity modulation *in vitro* and *in vivo* in prostate cancer cells [22, 23]. Therefore, this construct was used as a reporter system in the androgen-responsive LAPC-4 cells to assess the androgenic potential of RM-532-105. Using an increasing concentration of testosterone (T), we first demonstrated that the PSEBC-TSTA system can quantitatively assess AR activity (Fig 3) and that the reporter system can detect AR activation at castrated level of T (100 pg/mL; clinical castration level is 200 pg/mL). Interestingly, the addition of RM-532-105 to castrated levels of T does not significantly modulate AR transcriptional androgenic activity in LAPC-4 cells. Indeed, no statistically significant induction of the PSEBC-TSTA activity was observed upon treatment with the three doses of RM-532-105 tested, compared to T alone. Taken together, these results show that RM-532-105 mainly acts as a pure enzyme inhibitor.

### Effect of RM-532-105 on human LAPC-4 xenografts in nude mice

**Fig 4:**
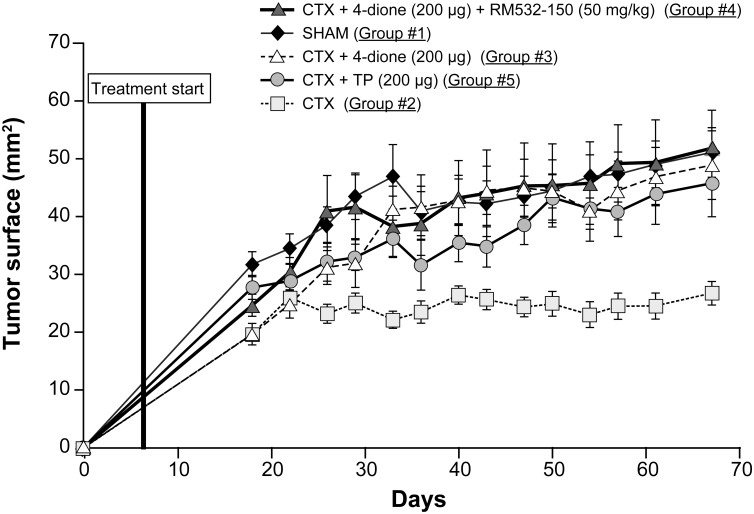
Tumor growth of human LAPC-4 cells xenografted in nude mice. Sham and castrated (CTX) mice received only the vehicle daily, whereas all the other castrated mice received daily s.c. 4-dione (200 μg), TP (200 μg) or 4-dione (200 μg) + RM-532-105 (50 mg/kg body weight) for 63 days. Data represent the mean ± SEM.

The *in vitro* results discussed above prompted us to use the LAPC-4 cells for our proof of concept in mice. As seen in Fig 4, all the tumor growth curves increased gradually during the experiment, except for Group 2. In fact, tumors in the castrated group (#2) were the smallest of all and displayed a sluggish and steady growth throughout the experiment. Conversely, the 4-dione-stimulated castrated group (#3) showed a sustained tumor growth profile, demonstrating that the tumors were sensitive to androgens synthesized from the inactive steroid (4-dione) [10] administered to mice. The tumor growth in this group was very similar to those of the non-castrated sham group (#1) and of the testosterone propionate- (TP) treated group (#5). In these groups, the androgens are produced from testis (group #1) and from the pro-drug TP (group #5), respectively. Surprisingly, the mice treated with the 17β-HSD3 inhibitor RM-532-105 (group #4) did not show any significant tumor decrease compared to group #3.

Following the LAPC-4 xenograft experiment, the mouse’s blood was collected 3 h following the last injection and the concentration of plasma unconjugated steroid hormones and inhibitor RM-532-105 were measured by GC/MS and LC/MS-MS, respectively. The same measurements were also done on the tumors collected at necropsy. The inhibitor was present in the plasma and tumors at concentrations of 174 ± 16 ng/mL (0.3 μM) and 30,180 ± 1440 ng/g (~ 46 μM), respectively (Table 1).

**Table 1:** Plasma and tumor concentrations of RM-532-105 in LAPC-4 cells xenografted in nude mice.

Plasma	174 ± 16 ng/mL	0.3 μM
Tumor	30,180 ± 1440 ng/g	~ 46 μM

**Fig 5:**
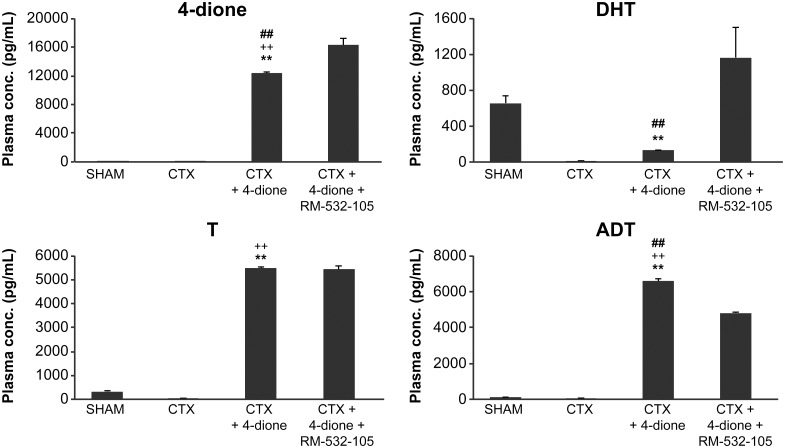
Plasma concentration of unconjugated steroids (4-dione, T, DHT and ADT) in LAPC-4 cells xenografted in nude mice and treated for 63 days. At the end of the xenograft experiment (see Fig 2) and 3 h after the last injection, the blood was collected and steroids measured by LC/MS-MS. CTX+4-dione group is significantly different (p<0.01) from the SHAM (**), CTX (^++^) or CTX+4-dione+RM-532-105 (^##^) groups.

**Fig 6:**
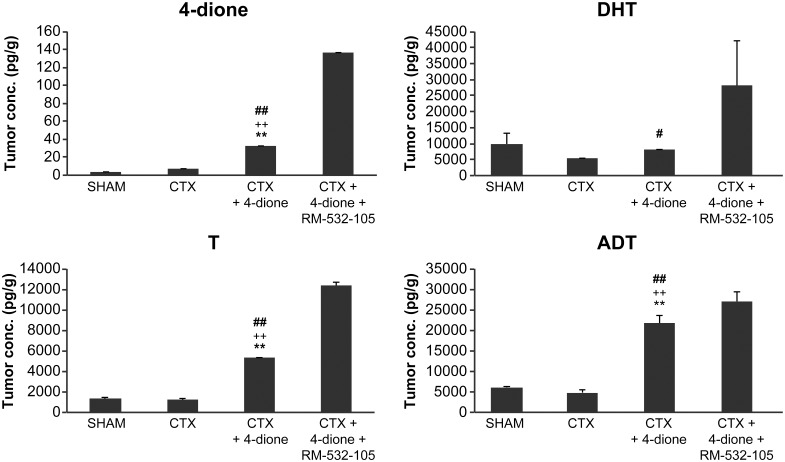
Tumor concentration of unconjugated steroids (4-dione, T, DHT and ADT) in LAPC-4 cells xenografted in nude mice and treated for 63 days. At the end of the xenograft experiment (see Fig 2), the tumors were collected and steroids measured by LC/MS-MS. CTX+4-dione group is significantly different (p<0.01) from the SHAM (**), CTX (^++^) or CTX+4-dione+RM-532-105 (^##^) groups. CTX+4-dione group is significantly different (p<0.05) from CTX+4-dione+RM-532-105 (^#^) group.

As shown in Figs 5 and 6, levels of 4-dione, T and ADT, but not DHT, increased in the plasma and tumors in mice treated with 4-dione, and were higher than those of SHAM and CTX mice. In the RM-532-105 treated group, 4-dione increased, thus showing its accumulation under the effect of a 17β-HSD3 blockade in both plasma and tumor. This effect was more pronounced in tumors, where the concentrations of steroids measured reflect the cumulative effect of treatments over 63 days. This is not the case for the plasma steroid concentration, which reflects the kinetics of RM-532-105 inhibitor 3 h after its administration. In plasma, the levels of T and DHT did not decrease significantly in mice treated with 4-dione and RM-532-105; to the contrary, DHT level increased when compared to the mice treated with 4-dione only (Fig 5). Similarly, in tumors treated with 4-dione and RM-532-105, levels of T, DHT and ADT were all increased (Fig 6). Clearly, the inhibition of 17β-HSD3 only cannot explain the unexpected increase of certain unconjugated steroids observed in tumors and plasma. It is important to note, however, that our analysis focused only on unconjugated steroids and did not include conjugated steroids such as glucuronides.

### Metabolism of 4-dione in LAPC-4 cells in the presence or absence of RM-532-105

**Fig 7:**
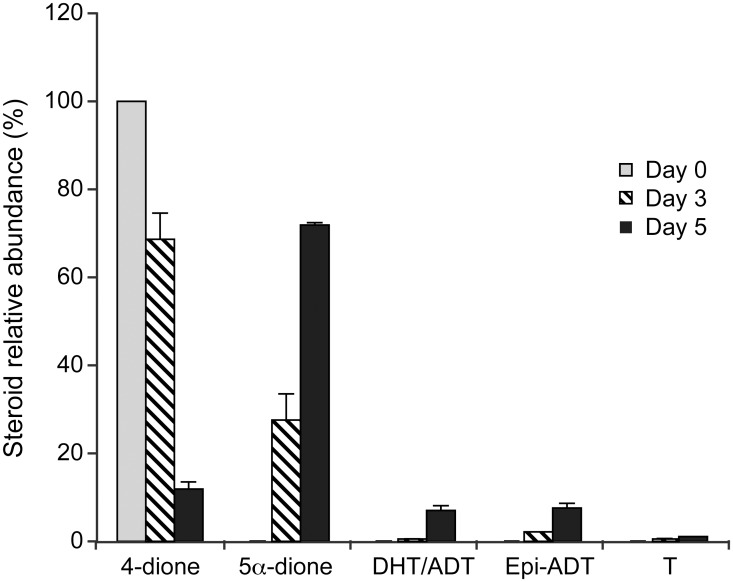
Metabolism of 4-dione in LAPC-4 cells. [^14^C]-4-dione (50 nM) was incubated with 20,000 LAPC-4 cells for 0, 3 and 5 days. Steroids were extracted from the culture medium, analyzed by TLC, and quantified.

The metabolism of 4-dione by LAPC-4 cells *in vitro* was studied using [^14^C]-labeled 4-dione. After incubation for 0, 3 and 5 days, steroids present as metabolites ([^14^C]-4-dione, [^14^C]-A-dione, [^14^C]-DHT/ADT, [^14^C]-epi-ADT and [^14^C]-T) were identified and quantified. As shown in Fig 7, the substrate [^14^C]-4-dione decreased over time and there is a corresponding formation of other steroids, especially A-dione that is the most abundant steroid formed, but also epi-ADT and the fraction corresponding to DHT and/or ADT. In that case, our TLC system cannot distinguish these two steroids. Surprisingly, only traces of T were detected.

**Fig 8:**
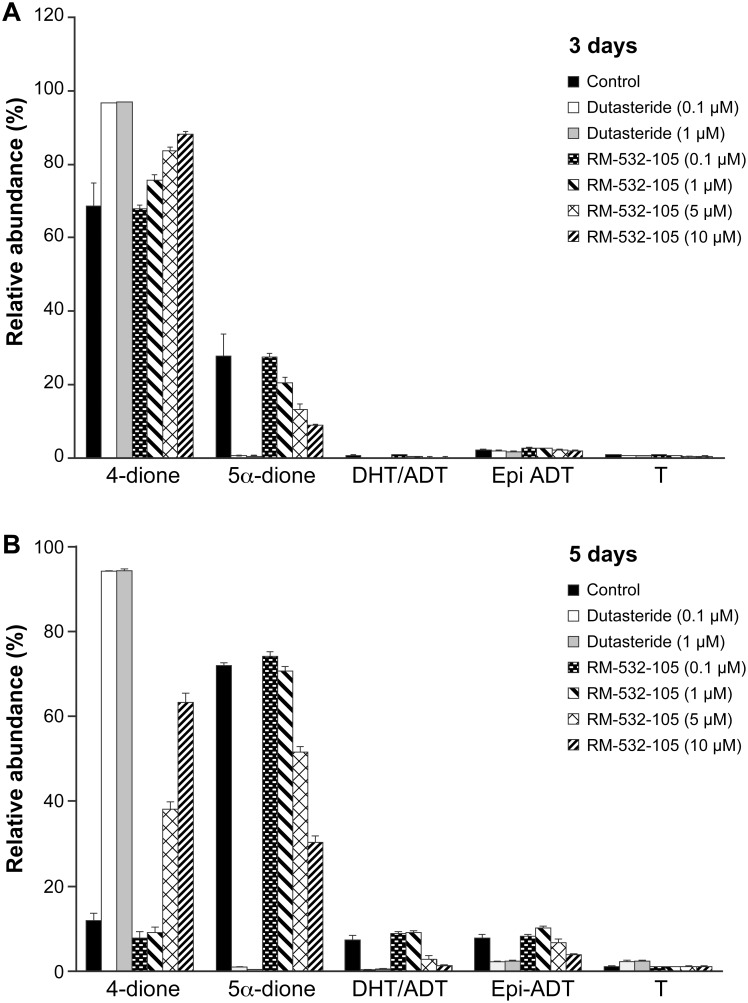
Metabolism of 4-dione in LAPC-4 cells in the presence of RM-532-105 or Dutasteride. [^14^C]-4-dione (50 nM) was incubated with 20,000 LAPC-4 cells in the absence or presence of RM-532-105 (0.1, 1, 5 and 10 μM) or Dutasteride (0.1 and 1 μM). After 3 days (**A**) or 5 days (**B**) of treatment, steroids were extracted from the culture medium, analyzed by TLC, and quantified.

We thereafter evaluated the effect of RM-532-105 at four concentrations on the 4-dione metabolism in LAPC-4 cells. We also tested 2 concentrations of Dutasteride, a potent inhibitor of 5α-reductase [24]. As displayed in Fig 8A and 8B, Dutasteride, both at 0.1 and 1 μM, blocked the metabolism of 4-dione. Similarly, but to a lesser extent, RM-532-105 also blocked the metabolism of 4-dione in a dose-dependent way. The effect was seen at both 3 and 5 days. As observed in the basal 4-dione metabolism assay (Fig 7), A-dione is the preferentially formed steroid. The production of T is clearly negligible, even in the presence of Dutasteride.

## Discussion

The compound RM-532-105 has already shown its efficacy in inhibiting the steroidogenic enzyme 17β-HSD3 *in vitro* (crude testis preparation and two transfected cell lines: HEK-293[17β-HSD3] and LNCaP[17β-HSD3]) [11,14]. After demonstrating that it reversed the LAPC-4 cell proliferation induced by 4-dione, the precursor of androgen T and DHT, we attempted to assess the *in vivo* inhibitory activity of RM-532-105 on LAPC-4 xenografts, a prostate cancer model. However, treating the castrated mice with the 17β-HSD3 inhibitor did not slow tumor growth produced by the 4-dione, the precursor of potent androgens. Rather, the T and DHT levels were increased within the tumors and DHT level was increased in plasma. This might be due to the administration of a supraphysiological level of 4-dione in castrated and stimulated mice that over-stimulated the tumor, making RM-532-105 action difficult. One hypothesis to explain the stable growth of tumors treated with 4-dione and RM-532-105 *vs* the control group could be a balance between the stimulatory effect of androgen (T and DHT) accumulation and the cytotoxic effect of RM-532-105 at higher concentration. In fact, no androgenicity was observed for RM-532-105 in two different assays using LAPC-4 cells (proliferation and transcriptional androgenic assay), but a cytotoxic effect was observed at a concentration over 2 μM in LAPC-4 cells and a concentration of RM-532-105 over 2 μM was measured in tumors.

The results mentioned above could be also coupled with a drug resistance in a castration-resistant prostate cancer (CRPC) pattern. In this situation, cancer cells undergo adaptation modification by acquiring an autonomous way of producing their own androgens by an intracrine pathway within the tumor, or by changes in androgen receptor gene amplification and mutation. Even more impressive, the synthesis of the most potent androgen DHT can bypasses the T pathway [25–28].

**Fig 9:**
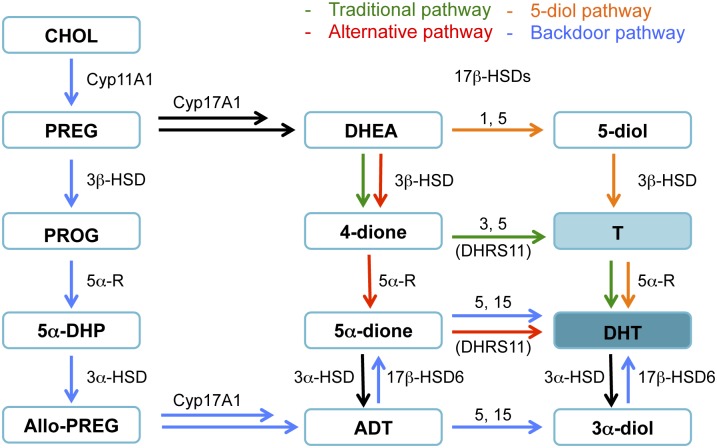
Representation of the different pathways involved in androgen formation. CHOL: cholesterol; PREG: pregnenolone; PROG: Progesterone; 5α-DHP: 5α-dihydroprogesterone; Allo-PREG: allopregnanolone; DHEA: dehydroepiandrosterone; 4-dione: 4-androstene-3,17-dione; 5α-dione: 5α-androstane-3,17-dione; ADT: androsterone; 5-diol: 5-androstene-3β,17β-diol; T: testosterone; DHT: dihydrotestosterone; 3α-diol: 5α-androstane-3α,17β-diol; Cyp17A1: cytochrome P45017A1; 3β-HSD: 3β-hydroxysteroid dehydrogenase/Δ^5^-Δ^4^-isomerase); 5α-R: 5α-reductase; 3α-HSD: 3α-hydroxysteroid dehydrogenase/Δ^5^-Δ^4^-isomerase; 17β-HSD: 17β-hydroxysteroid dehydrogenase; DHSR11: human dehydrogenase/reductase member 11.

Apart from the *traditional* DHT synthesis pathway (4-dione → T → DHT), 3 other pathways could be exploited by the tumor in CRPC to generate potent androgens, from the presence of precursors after androgen-deprivation therapy (Fig 9). Namely, the *alternative (5α-dione)* pathway (4-dione → 5α-dione → DHT) [8, 29–31], the *backdoor* pathway (progesterone → 5α-dihydroprogesterone → allopregnanolone → ADT → 3α-diol → DHT) [29, 31] and the 5-diol pathway (DHEA → 5-diol → T → DHT) [30]. In these pathways, in addition to 17β-HSD3, two other reductive 17β-HSD isoforms are involved (17β-HSD5 and 17β-HSD15). Thus, 17β-HSD5 catalyzes the transformation of 4-dione in T, 5α-dione in DHT, ADT in 3α-diol and DHEA in 5-diol [30], whereas 17β-HSD15 catalyzes the transformation of 5α-dione into DHT and ADT into 3α-diol [8, 31]. However, given the fact that the 17β-HSD5 protein is not observed in LAPC-4 cells [32], the way to produce DHT directly from 4-dione could involve the 17β-HSD15. Moreover, a 17β-HSD known as human dehydrogenase/reductase member 11 (DHRS11) has been recently reported [33]. Among its multiple functions, this reductive enzyme is able to transform 4-dione and 5α-dione into T and DHT, respectively. DHRS11 is also found in testis and could be involved in androgen production. Finally, the conversion of 3α-diol into DHT would be mainly catalyzed by 17β-HSD6 [34–36], and this oxidative enzyme overexpressed in prostate cancer patients undergoing androgen deprivation therapy could be another candidate in the androgen (DHT) synthesis [37].

Taking into account the fact that LAPC-4 cells are derived from a patient who went through androgen-deprivation therapy [20, 38], it is possible that in the model we used for our xenograft experiment (Fig 4), the presence and activity of 17β-HSD15 and/or DHRS11 could have prevented us to see the expected tumor regression in the group treated with RM-532-105. The presence of these 17β-HSDs could also explain the increased DHT levels in 4-dione stimulated groups (#3 and #4), where the administered 4-dione was used as a raw material by tumors to generate more DHT. Another potential explanation for the unexpected increase of certain steroids could be a potential inhibitory activity of RM-532-105 for steroidogenenic enzymes downstream to 4-dione, T and DHT, such as 3α-HSD and/or UGT2B7, UBT2B15 and UGT2B17 [39, 40], but this hypothesis remains to be validated.

Studying the metabolism of 4-dione in LAPC-4 cells, the steroid 5α-dione was preferentially formed over T, suggesting that the 17β-HSD3 or 17β-HSD5 involved in the transformation of 4-dione **→** T was not required. Rather, it was 5α-reductase that was engaged in the first metabolic step. These results were very similar to those obtained by Chang *et al*. [29] in LAPC-4 cells, where 4-dione was rapidly converted into 5α-reduced steroids instead of T. In that case, the 5α-reductase type 1 (SRD5A1) was identified as the dominant actor over the SRD5A2. They also reported an increased expression of SRD5A1 over SRD5A2 (the dominant isoenzyme expressed in the prostate) during progression to CRPC. This brings another level of complexity in LAPC-4 as a cell model for testing 17β-HSD3 inhibitors. It should be noted that by blocking the 4-dione metabolism, RM-532-105 proved to possess a SRD5A inhibitory activity, though less than Dutasteride, which is an inhibitor of both SRD5A1 and SRD5A2 [24]. However, the weak SRD5A inhibitory activity of RM-532-105 did not reduce *in vivo* the LAPC-4 tumor growth induced by 4-dione.

## Conclusion

This study was designated to assess *in vivo* the efficiency of RM-532-105, a 17β-HSD3 inhibitor. Using the LAPC-4 (AR^+^) xenograft model, we succeeded in stimulating tumor growth in castrated mice by using 4-dione, the precursor of androgen T, but the inhibitor RM-532-105 did not reduce their growth. Rather, although it was present in the blood of mice and very concentrated inside the tumors, levels of the androgens T and DHT were increased. In the process of finding the reason, we realized that 5α-reductase (SRD5A) instead of 17β-HSDs 3 and 5 is the predominant enzyme that metabolizes 4-dione in LAPC-4 cells, yielding 5α-dione and not T. 5α-dione is probably next converted into the most potent androgen DHT by a reductive isoform of 17β-HSD such as 17β-HSD15 or DHRS11. The former enzyme could be also involved in the conversion of ADT into 3α-diol, which could be oxidized into DHT by 17β-HSD6. Therefore, making a combination of SRD5A and 17β-HSD inhibitors could be another approach to evaluate this model for prostate cancer study, which is obviously complex. It is however important to mention that there is currently no clear consensus on which 17β-HSD isoforms, if any, regulate the three pathways potentially involved in the synthesis of potent androgen DHT. Therefore, it is also possible that a 17β-HSD isoform not discussed above could explain, at least in part, the fact that the tumor escapes from 17β-HSD3 inhibition.

In addition to these useful data relative to prostate cancer model (LAPC-4 xenografts), we fortuitously came across two interesting inputs during our studies. We discovered that RM-532-105 possesses a 5α-reductase inhibitory activity and besides, due to its accumulation inside the tumor, it can be an asset to develop a new generation of radiolabeling or radiotherapeutic agents for prostate cancer. Further investigations will however be necessary to determine whether this 17β-HSD3 inhibitor will preferentially accumulate in tumor tissues over normal tissues or could be used in combination with another inhibitor of an enzyme involved in DHT synthesis.
